# Supplementary material for: Sex Differences in Cancer Incidence Rates by Race and Ethnicity: Results from the Surveillance, Epidemiology, and End Results (SEER) Registry (2000–2019)
Source: Cancers (Basel). 2024 Feb 29;16(5):989. doi: 10.3390/cancers16050989 (PMC10930733; doi:10.3390/cancers16050989)
Supplement: Supplementary file 1 [file cancers-16-00989-s001.zip › cancers-2864461-supplementary.pdf]

Table S1. *ICD-O-3* codes and SEER recodes of cancer sites and selected histological subtypes.

Cancer Sites	*ICD-O-3* Site	*ICD-O-3* Histology (Type)
**Lip**	C000-C009	
**Tongue**	C019-C029	
**Salivary Gland**	C079-C089	
**Floor of Mouth**	C040-C049	
**Gum and Other Mouth**	C030-C039, C050-C059, C060-C069	
**Nasopharynx**	C110-C119	
**Tonsil**	C090-C099	
**Oropharynx**	C100-C109	
**Hypopharynx**	C129, C130-C139	
**Other Oral Cavity and Pharynx**	C140, C142, C148	
**Esophagus**	C150-C159	
**Stomach**	C160-C169	
**Small Intestine**	C170-C179	
**Colon and Rectum (Including Cecum, Appendix, Ascending Colon, Hepatic Flexure, Transverse Colon, Splenic Flexure, Descending Colon, Sigmoid Colon, Large Intestine, Rectosigmoid Junction, and Rectum)**	C180-C189, C199, C209, C260	excluding 9050-9055, 9140, 9590-9993
**Anus, Anal Canal, and Anorectum**	C210-C212, C218	
**Liver**	C220	
**Gallbladder**	C239	
**Other Biliary**	C240-C249	
**Pancreas**	C250-C259	
**Retroperitoneum**	C480	
**Peritoneum, Omentum, and Mesentery**	C481-C482	
**Other Digestive Organs**	C268-C269, C488	
**Nose, Nasal Cavity, and Middle Ear**	C300-C301, C310-C319	
**Larynx**	C320-C329	
**Lung and Bronchus**	C340-C349	
**Pleura**	C384	
**Trachea, Mediastinum, and Other Respiratory Organs**	C339, C381-C383, C388, C390, C398, C399	
**Bones and Joints**	C400-C419	
**Soft Tissue Including Heart**	C380, C470-C479, C490-C499	
**Skin Excluding Basal and Squamous**	C440-C449	excluding 8000-8005, 8010-8046, 8050-8084, 8090-8110, 8720-8790, 9050-9055, 9140, 9590-9993

**Melanoma of the Skin**	C440-C449	8720-8790
**Other Non-Epithelial Skin**	C440-C449	excluding 8000-8005, 8010-8046, 8050-8084, 8090-8110, 8720-8790, 9050-9055, 9140, 9590-9993
**Urinary Bladder**	C670-C679	
**Kidney and Renal Pelvis**	C649, C659	
**Ureter**	C669	
**Other Urinary Organs**	C680-C689	
**Eye and Orbit**	C690-C699	excluding 9050-9055, 9140, 9590-9993
**Brain**	C710-C719	
**Cranial Nerves Other Nervous System**	C700-C729	
**Thyroid**	C739	
**Other Endocrine Including Thymus**	C379, C740-C749, C750-C759	
**Hodgkin Lymphoma (Including Nodal and Extranodal Hodgkin Lymphoma)**	C024, C098-C099, C111, C142, C379, C422, C770-C779	9650-9667
**Non-Hodgkin Lymphoma (Including Nodal and Extranodal Non-Hodgkin Lymphoma)**	C024, C098, C099, C111, C142, C379, C422, C770-C779	9590-9597, 9670-9671, 9673, 9675, 9678-9680, 9684, 9687-9691, 9695, 9698-9702, 9705, 9708-9709, 9712, 9714-9719, 9724-9729, 9735, 9737-9738, 9811-9818, 9823, 9827, 9837
**Myeloma**		9731-9732, 9734
**Lymphocytic Leukemia (Including Acute, Chronic, and Other Lymphocytic Leukemia)**	C420, C421, C424	9811-9819, 9820, 9823, 9826, 9832-9837, 9940
**Myeloid and Monocytic Leukemia (Including Acute Myeloid Leukemia, Acute Monocytic Leukemia, Chronic Myeloid Leukemia, and Other Myeloid and Monocytic Leukemia)**		9840, 9860-9861, 9863, 9865-9867, 9869, 9871-9879, 9891, 9895-9897, 9898, 9910-9912, 9920, 9930, 9945-9946,
**Other Leukemia (Including Other Acute Leukemia, Aleukemic, Subleukemic, and NOS)**		9733, 9742, 9800-9801, 9805-9809, 9827, 9870, 9931, 9948, 9963-9964
**Mesothelioma**		9050-9055
**Kaposi Sarcoma**		9140
Cancer site and histology type		
**Esophagus Adenocarcinoma**	C150-C159	8140-8231, 8250-8551, 8570-8574, 8576
**Esophagus SCC**	C150-C159	8050-8078, 8083-8084
**Gastric Cardia Adenocarcinoma**	C160	8140-8231, 8250-8551, 8570-8574, 8576
**Gastric Non-Cardia Adenocarcinoma**	C161-C169	8140-8231, 8250-8551, 8570-8574, 8576
**Liver HCC**	C220	8170-8175
**Liver ICC**	C220	8160/3

**Lung and Bronchus Adenocarcinoma**	C340-C349	8140-8231, 8250-8551, 8570-8574, 8576
**Lung and Bronchus SCC**	C340-C349	8002, 8040-8045, 8240, 8246
**Lung and Bronchus Small Cell Carcinoma**	C340-C349	8002, 8040-8045, 8240, 8246
**Lung and Bronchus Large Cell Carcinoma**	C340-C349	8012
**Urinary Bladder TCC**	C670-C679	8120-8131

Abbreviations: *ICD-O-3, International Classification of Diseases for Oncology, 3rd Edition*; WHO, World Health Organization; NOS, not otherwise specified; SCC, squamous cell carcinoma; HCC, hepatocellular carcinoma; ICC, intrahepatic cholangiocarcinoma; TCC, transitional cell carcinoma.

Table S2. Male and female incidence rates per 100,000 by racial and ethnic group, SEER 2000-2019.

	**Non-Hispanic White**		**Non-Hispanic Black**		**Non-Hispanic Asian Pacific Islander**		**Latino**		**Non-Hispanic American Indian/Alaskan Native**	
	**Male rate**	**Female rate**	**Male rate**	**Female rate**	**Male rate**	**Female rate**	**Male rate**	**Female rate**	**Male rate**	**Female rate**
**Lip**	2.6	0.7	0.2	0.1	0.2	0.1	1.0	0.2	1.3	0.3
**Tongue**	7.5	2.8	5.1	1.6	3.6	2.1	3.4	1.6	4.0	1.6
**Salivary Gland**	2.5	1.4	1.7	1.4	1.5	1.4	1.5	1.1	1.7	1.5
**Floor of Mouth**	1.4	0.6	1.5	0.5	0.4	0.2	0.8	0.3	1.5	0.6
**Gum and Other Mouth**	2.7	2.0	2.8	1.7	2.1	1.3	1.7	1.2	2.5	1.4
**Nasopharynx**	0.7	0.3	1.4	0.5	6	2.1	0.7	0.3	4.8	2.0
**Tonsil**	4.4	1.0	4.3	0.9	1.5	0.3	2.5	0.5	2.6	0.5
**Oropharynx**	0.9	0.3	1.5	0.4	0.3	0.1	0.6	0.1	0.8	0.2
**Hypopharynx**	1.5	0.4	2.5	0.5	1.3	0.2	1.3	0.2	2.6	0.4
**Other Oral Cavity and Pharynx**	0.5	0.2	0.7	0.2	0.2	0.0	0.3	0.1	0.6	0.2
**Esophagus**	10.9	2.7	11.1	3.9	6.1	1.4	7.3	1.5	10.8	3.5
**Stomach**	12.1	5.4	22.4	11.8	24.5	13.7	21.3	13.1	28.4	14.8
**Small Intestine**	3.5	2.4	5.5	4.2	2.1	1.3	2.6	1.9	2.7	1.9
**Colon and Rectum**	72.7	55.5	86.7	66.9	67.1	47.0	61.1	43.4	86.8	71.0
**Anus, Anal Canal, and Anorectum**	2.2	3.0	3.5	2.3	0.7	0.7	1.4	1.9	1.8	2.1
**Liver**	11.5	4.1	21.9	6.8	30	11.0	25.4	9.8	28.1	14.3
**Gallbladder**	0.9	1.6	1.5	2.4	1.6	2.0	1.8	4.5	4.7	6.7
**Other Biliary**	2.7	1.8	2.8	2.0	4.2	2.6	4.0	3.1	4.8	4.2
**Pancreas**	19.1	14.5	23.8	20.4	16.1	12.9	16.4	14.3	20.8	15.7

**Retroperitoneum**	0.6	0.5	0.5	0.6	0.5	0.5	0.6	0.5	0.2	0.4
**Peritoneum, Omentum, and Mesentery**	0.1	1.5	0.1	0.8	0.1	0.9	0.2	1.2	0.1	0.8
**Other Digestive Organs**	1	0.7	1.3	1.0	1	0.7	1.1	1	2.5	2.3
**Nose, Nasal Cavity, and Middle Ear**	1.2	0.7	1.3	0.7	1.1	0.6	1.0	0.7	1.7	1.1
**Larynx**	7.9	1.7	12.4	2.3	3.6	0.4	6.5	0.8	6.3	1.1
**Lung Bronchus**	96.1	70.9	126.3	69.0	75.8	41.3	54.0	33.3	89.1	57.6
**Pleura**	0.1	0.0	0.0	0.0	0	0.0	0.1	0	0.1	0.0
**Trachea, Mediastinum, and Other Respiratory Organs**	0.3	0.1	0.3	0.2	0.3	0.1	0.3	0.1	0.4	0.0
**Soft Tissue Including Heart**	1.2	0.9	0.9	0.7	0.7	0.5	0.9	0.7	1.0	1.2
**Skin Excluding Basal and Squamous**	5.2	3.3	4.9	3.9	3.8	2.7	4.4	3.4	4.8	3.3
**Melanoma of the Skin**	52.1	34.0	1.9	1.2	2.2	1.8	6.9	6.6	7.0	6.9
**Other Non-Epithelial Skin**	4.7	2.3	1.8	1.6	1.5	1.1	1.7	1.5	1.7	1.9
**Urinary Bladder**	56.9	13.7	30.0	9.9	23.8	5.9	26.7	7.1	23.2	5.1
**Kidney and Renal Pelvis**	26.2	12.4	31.8	15.0	16.2	7.5	26.2	14.6	44.8	22.4
**Ureter**	1.2	0.5	0.5	0.3	1	0.6	0.7	0.3	0.5	0.1
**Other Urinary Organs**	0.8	0.2	0.9	0.5	0.5	0.2	0.5	0.2	0.6	0.1
**Eye and Orbit**	1.6	1.2	0.3	0.2	0.3	0.2	0.8	0.5	0.4	0.6
**Brain**	10.7	7.1	5.3	3.6	4.6	3.1	6.8	5	6.0	3.5
**Cranial Nerves Other Nervous System**	0.5	0.4	0.5	0.4	0.4	0.4	0.4	0.4	0.3	0.5
**Thyroid**	8.7	22.8	4.2	13.3	7.6	23.6	6.2	21.8	6.6	21.5
**Other Endocrine Including Thymus**	0.8	0.7	1.1	1.1	1.2	0.9	0.7	0.6	0.7	0.3
**Hodgkin Lymphoma**	4.3	3.4	4.0	2.9	1.7	1.3	3.4	2.3	1.7	1.5

**Non-Hodgkin Lymphoma**	36.2	23.8	26.8	17.3	23.8	16.1	28.0	20.7	21.5	18.1
**Myeloma**	10.6	6.3	22.1	15.9	6.6	4.5	9.9	6.8	10.2	8.6
**Lymphocytic Leukemia**	13.4	6.8	8.7	4.4	3.4	1.9	6.2	3.9	5.3	3.1
**Myeloid and Monocytic Leukemia**	10.7	6.6	8.9	6.3	8.3	5.2	8.2	5.6	7.6	4.7
**Other Leukemia**	1.3	0.8	1.3	1.0	0.9	0.6	0.9	0.6	1.4	0.9
**Mesothelioma**	2.7	0.6	1.4	0.3	0.9	0.3	2.4	0.6	2.2	0.7
**Kaposi Sarcoma**	3.6	0.1	7.1	0.3	0.9	0.0	3.7	0.3	2.1	0.3

Table S3. Male and female incidence rates per 100,000 select histological cancer subtypes by racial and ethnic group, SEER 2000-2019.

	**Non-Hispanic White**		**Non-Hispanic Black**		**Non-Hispanic Asian Pacific Islander**		**Latino**		**Non-Hispanic American Indian/Alaskan Native**	
	**Male rate**	**Female rate**	**Male rate**	**Female rate**	**Male rate**	**Female rate**	**Male rate**	**Female rate**	**Male rate**	**Female rate**
Esophagus Adenocarcinoma	2.3	1.4	8.6	3.2	4.2	1.1	2.7	0.7	5.1	1.7
Esophagus SCC	7.7	1.0	1.6	0.5	1.4	0.2	3.8	0.5	4.6	1.4
Gastric Cardia Adenocarcinoma	5.7	1.3	3.1	1.1	3.7	1.1	3.8	1.3	6.0	2.0
Gastric Non-Cardia Adenocarcinoma	7.7	5.0	20.9	11.6	22.4	13.9	19.5	13.2	23.8	14.2
Liver HCC	10.2	3.0	20.7	5.8	27.8	9.4	23.6	8.2	24.8	12.0
Liver ICC	1.6	1.1	1.4	1.1	2.4	1.7	2.0	1.7	3.3	2.4
Lung and Bronchus Adenocarcinoma	32.4	29.3	42.4	29.0	32.5	24.9	19.2	14.9	21.0	17.1
Lung and Bronchus SCC	21.8	10.2	31.6	11.9	14.7	3.8	11.4	4.6	25.5	12.6
Lung and Bronchus Small Cell Carcinoma	13.2	12.1	12.4	8.9	7.7	3.1	6.6	4.7	13.7	10.8
Lung and Bronchus Large Cell Carcinoma	2.8	1.9	4.6	1.9	1.7	0.7	1.2	0.7	1.2	0.7
Urinary Bladder TCC	53.8	12.6	27.1	8.5	22.4	5.4	24.8	6.2	20.7	3.8

Abbreviations: MF IRR, male-to-female incidence rate ratio; CI, confidence interval; SCC, squamous cell carcinoma; HCC, hepatocellular carcinoma; ICC, intrahepatic cholangiocarcinoma; TCC, transitional cell carcinoma.

**Table S4.** Male and female incidence rates per 100,000 by 10-year age groups among non-Hispanic White individuals, SEER 2000-2019.														
	**20–29 years**		**30–39 years**		**40–49 years**		**50–59 years**		**60–69 years**		**70–79 years**		**80+ years**	
**Cancer Site**	**Male rate**	**Female rate**	**Male rate**	**Female rate**	**Male rate**	**Female rate**	**Male rate**	**Female rate**	**Male rate**	**Female rate**	**Male rate**	**Female rate**	**Male rate**	**Female rate**
**Lip**	0.1	0.0	0.4	0.1	1.1	0.3	2.2	0.6	4.3	1.1	7.9	1.9	10.4	3.0
**Tongue**	0.3	0.2	0.9	0.6	3.8	1.5	11.1	3.3	17.3	5.2	15.1	6.5	10.4	6.7
**Salivary Gland**	0.3	0.4	0.4	0.6	0.9	0.8	1.9	1.2	3.8	2.0	6.4	2.9	11.2	3.8
**Floor of Mouth**	0.0	0.0	0.1	0.0	0.6	0.2	2.0	0.7	3.2	1.2	2.9	1.7	1.9	1.4
**Gum and Other Mouth**	0.1	0.2	0.3	0.3	1.0	0.6	2.7	1.6	4.8	3.1	6.9	5.6	8.4	8.7
**Nasopharynx**	0.1	0.0	0.2	0.1	0.5	0.2	1.1	0.3	1.4	0.5	1.2	0.6	1.1	0.6
**Tonsil**	0.0	0.0	0.3	0.1	3.7	0.6	9.4	1.6	10.0	2.2	5.9	2	3.0	1.0
**Oropharynx**	0.0	0.0	0.0	0.0	0.4	0.1	1.4	0.3	2.3	0.6	1.9	0.6	1.3	0.6
**Hypopharynx**	0.0	0.0	0.0	0.0	0.5	0.1	1.9	0.4	3.3	0.9	4.2	1.3	2.7	0.8
**Other Oral Cavity and Pharynx**	0.0	0.0	0.0	0.0	0.2	0.1	0.6	0.2	1.0	0.3	1.1	0.6	1.2	0.5
**Esophagus**	0.1	0.0	0.5	0.1	2.6	0.5	10.2	2.1	24.8	5.2	33.9	9.7	35.2	12.3
**Stomach**	0.2	0.2	0.9	0.6	3.2	1.7	9.4	3.9	22.1	8.4	38.8	17	52.5	28.7
**Small Intestine**	0.1	0.1	0.4	0.4	1.4	1.1	3.4	2.6	6.0	4.4	8.5	6.2	9.4	6.7
**Colon and Rectum**	1.7	1.8	6.5	5.8	21.8	18.5	64.6	45.4	127.8	88.1	218.9	174.6	294.5	273.4
**Anus, Anal Canal, and Anorectum**	0.0	0.0	0.4	0.3	1.6	1.9	3.1	4.4	3.9	6.4	4.2	6.6	4.6	6.2
**Liver**	0.2	0.2	0.5	0.4	3.0	1.0	15.6	3.8	28.6	8.3	31.1	13.3	30.7	16.6
**Gallbladder**	0.0	0.0	0.1	0.1	0.2	0.3	0.5	1.0	1.5	2.8	3.2	6.2	5.3	9.0
**Other Biliary**	0.0	0.0	0.2	0.1	0.5	0.3	1.7	1.2	4.5	3.0	8.7	6.1	14.3	10.1
**Pancreas**	0.1	0.2	0.8	0.7	4.1	2.8	15.4	9.9	37.4	26.6	62.2	51.9	81.7	76.4
**Retroperitoneum**	0.1	0.1	0.2	0.2	0.3	0.3	0.6	0.6	1.0	0.9	1.5	1.3	1.7	1.1
**Peritoneum, Omentum, and Mesentery**	0.0	0.0	0.0	0.1	0.0	0.5	0.1	1.4	0.2	3.7	0.3	5.4	0.4	3.7
**Other Digestive Organs**	0.0	0.0	0.1	0.1	0.2	0.2	0.7	0.5	1.6	1.2	3.2	2.3	4.9	4.2

**Nose, Nasal Cavity, and Middle Ear**	0.1	0.1	0.3	0.2	0.5	0.4	1.1	0.7	2.2	1.2	2.9	1.8	3.8	2.4
**Larynx**	0.1	0.0	0.3	0.2	2.3	0.7	9.0	2.3	19.0	4.0	23.4	4.7	20.5	2.9
**Lung Bronchus**	0.4	0.4	2.1	2.3	14.1	13.9	64.6	55.1	195.8	150.5	351.0	267.5	362.9	237.7
**Pleura**	0.0	0.0	0.0	0.0	0.0	0.0	0.0	0.0	0.1	0.0	0.2	0.1	0.5	0.2
**Trachea, Mediastinum, and Other Respiratory Organs**	0.3	0.0	0.2	0.0	0.2	0.1	0.3	0.1	0.4	0.2	0.4	0.3	0.6	0.3
**Soft Tissue Including Heart**	1.0	0.8	0.7	0.6	0.8	0.8	1.1	0.8	1.4	1.0	1.6	1	2.2	1.6
**Skin Excluding Basal and Squamous**	1.4	1.1	2.0	1.4	2.8	2.0	4.5	3.1	7.2	4.8	10.8	6.7	15.8	8.5
**Melanoma of the Skin**	6.1	12.2	15.2	22.6	28.3	30.2	50.2	36.7	82.2	44.8	109.1	47.7	127.5	49.7
**Other Non-Epithelial Skin**	0.5	0.6	0.8	0.9	1.4	1.1	2.3	1.5	5.3	2.8	11.6	5.3	24.3	9.7
**Urinary Bladder**	0.6	0.3	2.2	0.8	9.0	2.7	32.4	9.5	95.9	24.8	190.1	47.4	266.1	64.1
**Kidney and Renal Pelvis**	0.6	0.5	3.2	2.1	11.4	5.7	28.0	13.2	51.1	23.6	65.0	33.6	61.3	33.3
**Ureter**	0.0	0.0	0.0	0.0	0.1	0.0	0.4	0.2	1.6	0.7	3.3	1.8	4.7	2.8
**Other Urinary Organs**	0.0	0.0	0.0	0.0	0.1	0.0	0.3	0.2	0.9	0.4	1.8	0.6	3.7	1.2
**Eye and Orbit**	0.1	0.2	0.4	0.4	0.9	0.7	1.9	1.4	2.9	2.3	3.9	2.7	4.5	2.7
**Brain**	3.4	2.7	4.7	3.5	6.6	4.5	11.7	7.4	18.0	11.8	22.8	16.6	22.1	15.3
**Cranial Nerves Other Nervous System**	0.2	0.2	0.3	0.3	0.4	0.4	0.5	0.5	0.6	0.6	0.8	0.6	0.6	0.6
**Thyroid**	2.9	14.0	6.0	23.7	8.0	25.8	10.5	25.9	12.1	21.8	10.2	16.2	6.8	9.1
**Other Endocrine Including Thymus**	0.3	0.2	0.4	0.3	0.6	0.5	1.0	0.8	1.3	1.2	1.5	1.2	1.3	1.1
**Hodgkin Lymphoma**	5.5	5.7	4.3	3.9	3.4	2.2	3.0	1.7	3.3	2.1	3.9	2.9	4.1	2.8
**Non-Hodgkin Lymphoma**	3.8	2.4	9.5	4.9	16.7	10.0	31.6	21.2	57.5	41.6	94.7	71.5	118.6	84.0
**Myeloma**	0.1	0.0	0.6	0.3	2.5	1.8	8.5	5.3	20.1	13.0	34.3	22	43.7	26.4
**Lymphocytic Leukemia**	0.8	0.5	1.4	0.7	4.0	2.0	11.9	5.9	25.5	13.0	39.6	22.2	51.6	29.6

**Myeloid and Monocytic Leukemia**	1.5	1.5	2.4	1.9	3.6	2.8	6.5	4.8	14.6	8.7	29.6	16.5	41.5	24.5
**Other Leukemia**	0.1	0.1	0.2	0.1	0.2	0.2	0.5	0.4	1.6	1.0	3.9	2.4	9.1	6.2
**Mesothelioma**	0.0	0.0	0.1	0.1	0.2	0.2	1.2	0.5	4.3	1.0	10.4	2.1	15.1	2.5
**Kaposi Sarcoma**	1.3	0.0	6.7	0.1	5.2	0.0	2.2	0.0	1.2	0.1	1.3	0.3	2.4	1.0

Table S5. Male and female incidence rates per 100,000 by 10-year age groups among non-Hispanic Black individuals, SEER 2000-2019.

	20–29 years		30–39 years		40–49 years		50–59 years		60–69 years		70–79 years		80+ years	
**Cancer Site**	**Male rate**	**Female rate**	**Male rate**	**Female rate**	**Male rate**	**Female rate**	**Male rate**	**Female rate**	**Male rate**	**Female rate**	**Male rate**	**Female rate**	**Male rate**	**Female rate**
**Lip**	0.0	0.0	0.0	0.1	0.1	0.1	0.2	0.1	0.3	0.2	0.5	0.2	1.4	0.3
**Tongue**	0.1	0.0	0.5	0.3	3.0	1.1	8.1	2.5	12.1	2.7	9.2	3.0	7.2	2.7
**Salivary Gland**	0.4	0.4	0.4	0.8	1.2	1.0	1.8	1.3	2.5	2.2	3.4	1.6	5.0	2.9
**Floor of Mouth**	0.0	0.0	0.2	0.0	0.9	0.2	2.6	0.7	3.4	1.1	3.1	1.1	1.8	0.8
**Gum and Other Mouth**	0.1	0.2	0.4	0.3	1.5	0.9	3.7	2.2	5.3	2.7	5.0	4.3	6.4	4.8
**Nasopharynx**	0.3	0.1	0.5	0.2	1.5	0.4	1.7	0.4	1.8	0.8	2.5	1.2	1.3	0.3
**Tonsil**	0.0	0.0	0.3	0.1	3.1	0.7	7.7	1.4	9.0	2.3	7.3	1.7	5.1	0.4
**Oropharynx**	0.0	0.0	0.1	0.0	0.7	0.2	2.3	0.6	4.0	0.9	3.4	0.7	1.2	0.6
**Hypopharynx**	0.0	0.0	0.0	0.0	0.9	0.3	3.3	0.9	6.0	1.3	6.3	1.1	4.4	0.6
**Other Oral Cavity and Pharynx**	0.0	0.0	0.0	0.0	0.3	0.1	1.0	0.1	1.5	0.6	1.9	0.6	0.9	0.6
**Esophagus**	0.1	0.0	0.3	0.2	3.3	1.4	12.7	4.4	26.9	9.5	31.5	12.4	23.3	10.7
**Stomach**	0.4	0.3	1.7	1.5	6.0	4.0	16.9	9.2	38.6	17.7	68.6	37.0	93.5	64.3
**Small Intestine**	0.1	0.1	0.7	0.7	2.5	2.1	5.1	4.3	9.7	7.9	12.3	10.7	14.0	11.9
**Colon and Rectum**	1.6	1.4	7.0	7.2	28.2	24.8	86.8	68.9	163.4	124.6	236.2	200.2	289.6	273.7
**Anus, Anal Canal, and Anorectum**	0.3	0.0	1.8	0.3	4.0	1.5	5.0	3.6	3.7	4.4	3.5	4.3	4.0	4.9
**Liver**	0.4	0.2	1.5	0.5	6.4	1.8	32.6	8.5	66.4	18.1	49.8	19.2	38.5	22.5
**Gallbladder**	0.0	0.0	0.1	0.2	0.3	0.7	0.9	2.6	3.0	5.1	5.1	8.0	4.7	10.2
**Other Biliary**	0.0	0.0	0.2	0.1	0.6	0.5	1.9	1.5	5.6	4.0	8.3	7.2	12.9	9.5
**Pancreas**	0.1	0.2	1.1	0.9	5.6	4.3	22.9	15.9	49.5	40.1	70.9	73.6	88.8	105
**Retroperitoneum**	0.0	0.1	0.2	0.2	0.2	0.4	0.4	0.8	0.8	1.0	1.5	1.7	0.7	1.2
**Peritoneum, Omentum, and Mesentery**	0.0	0.0	0.0	0.0	0.1	0.1	0.0	0.7	0.4	2.0	0.1	2.8	0.6	1.8
**Other Digestive Organs**	0.0	0.0	0.2	0.1	0.3	0.2	1.0	0.6	2.2	2.0	3.9	2.9	4.4	5.5

**Nose, Nasal Cavity, and Middle Ear**	0.1	0.1	0.4	0.3	0.8	0.5	1.5	0.7	1.7	1.1	2.5	1.7	3.1	2.6
**Larynx**	0.1	0.0	0.4	0.2	3.7	0.9	15.1	3.4	31.3	6.5	31.4	5.5	28.1	4.0
**Lung Bronchus**	0.4	0.4	3.4	2.6	24.7	18.4	111	67	275.5	156.4	416.1	236.5	404.1	211.1
**Pleura**	0.0	0.0	0.0	0.0	0.0	0.0	0.0	0.1	0.0	0.1	0.1	0.1	0.0	0.2
**Trachea, Mediastinum, and Other Respiratory Organs**	0.2	0.1	0.1	0.0	0.2	0.1	0.3	0.2	0.4	0.2	0.2	0.5	0.8	0.4
**Soft Tissue Including Heart**	0.6	0.6	0.6	0.5	0.8	0.6	0.7	0.7	1.3	0.8	0.9	0.9	1.8	1.4
**Skin Excluding Basal and Squamous**	1.8	1.3	2.6	1.9	3.8	2.5	4.7	4.6	7.0	5.7	6.9	7.0	9.8	9.3
**Melanoma of the Skin**	0.1	0.2	0.3	0.6	0.5	0.6	1.4	0.9	2.9	1.6	4.5	3.1	7.0	4.1
**Other Non-Epithelial Skin**	0.7	0.9	1.2	1.2	1.4	1.6	1.5	1.2	1.7	1.5	2.8	2.3	3.1	2.9
**Urinary Bladder**	0.2	0.1	1.3	0.4	4.9	1.4	18.6	5.7	47.1	16.2	85.3	36.3	129.4	54.8
**Kidney and Renal Pelvis**	0.9	0.8	4.0	2.4	14.7	7.4	36.3	15.2	61.6	29.8	62.9	39.9	52.3	36.3
**Ureter**	0.0	0.0	0.0	0.0	0.0	0.0	0.1	0.1	0.9	0.2	1.4	1.0	2.4	1.8
**Other Urinary Organs**	0.0	0.0	0.0	0.0	0.2	0.2	0.3	0.5	1.2	1.1	2.2	1.3	3.0	1.7
**Eye and Orbit**	0.0	0.0	0.1	0.1	0.2	0.1	0.2	0.1	0.5	0.4	0.7	0.2	0.7	0.6
**Brain**	1.3	1.3	2.2	1.6	3.3	2.4	6.0	3.7	8.8	6.1	10.5	8.8	11.8	8.1
**Cranial Nerves Other Nervous System**	0.2	0.3	0.3	0.2	0.3	0.4	0.6	0.6	0.6	0.5	1.1	0.7	0.8	0.9
**Thyroid**	0.7	4.3	1.8	10.0	3.4	14.6	5.6	16.8	5.5	17.8	7.5	15.5	4.5	8.5
**Other Endocrine Including Thymus**	0.2	0.3	0.4	0.6	0.9	0.7	1.4	1.2	1.7	2.1	1.9	1.8	1.9	1.8
**Hodgkin Lymphoma**	3.9	3.9	4.0	3.3	4.3	2.4	3.7	2.4	3.0	2.0	3.0	2.2	1.6	1.4
**Non-Hodgkin Lymphoma**	4.8	3.0	11.6	5.7	19.8	10.9	28.8	18.1	41.4	28.9	50.7	43.8	52	47.4
**Myeloma**	0.1	0.0	1.6	1.1	6.1	5.9	21.1	16.2	44.1	33.0	65.1	54.8	76.4	58.3
**Lymphocytic Leukemia**	0.7	0.3	0.9	0.5	2.5	1.2	7.2	3.3	16.5	8.9	24.2	14.1	33.6	18.9

**Myeloid and Monocytic Leukemia**	1.7	1.4	2.7	2.2	4.4	3.0	5.9	4.9	11.7	8.7	20	13.8	28.4	22
**Other Leukemia**	0.2	0.1	0.3	0.2	0.5	0.4	0.9	0.8	2.1	1.2	2.8	2.7	6.2	5.0
**Mesothelioma**	0.0	0.0	0.0	0.1	0.2	0.1	0.7	0.2	2.2	0.6	4.8	1.4	7.4	1.4
**Kaposi Sarcoma**	5.2	0.1	13.8	0.4	9.6	0.5	3.8	0.3	1.7	0.1	1.5	0.4	1.9	0.5

Table S6. Male and female incidence rates per 100,000 by 10-year age groups among non-Hispanic Asian American/Pacific Islander individuals, SEER 2000-2019.

	**20–29 years**		**30–39 years**		**40–49 years**		**50–59 years**		**60–69 years**		**70–79 years**		**80+ years**	
**Cancer Site**	**Male rate**	**Female rate**	**Male rate**	**Female rate**	**Male rate**	**Female rate**	**Male rate**	**Female rate**	**Male rate**	**Female rate**	**Male rate**	**Female rate**	**Male rate**	**Female rate**
**Lip**	0.0	0.0	0.1	0.0	0.1	0.0	0.2	0.1	0.3	0.3	0.3	0.4	0.4	0.6
**Tongue**	0.2	0.1	0.9	0.7	1.7	1.1	4.1	2.3	6.6	3.6	8.8	4.7	9.1	6.6
**Salivary Gland**	0.4	0.5	0.5	0.7	1.1	1.1	1.5	1.4	2.6	2.0	3.3	2.1	3.9	2.5
**Floor of Mouth**	0.0	0.0	0.0	0.0	0.2	0.1	0.5	0.2	1.0	0.3	1.0	0.7	0.7	0.7
**Gum and Other Mouth**	0.1	0.2	0.3	0.3	0.8	0.5	2.0	0.9	3.8	2.0	5.4	3.7	6.4	6.6
**Nasopharynx**	0.7	0.5	3	1.4	7.7	2.5	9.6	3.2	9.6	3.0	7.6	2.3	4.9	2.5
**Tonsil**	0.0	0.0	0.2	0.1	0.9	0.2	2.3	0.4	3.8	0.9	3.1	0.5	2.2	0.7
**Oropharynx**	0.0	0.0	0.0	0.0	0.1	0.1	0.4	0.1	0.6	0.2	0.7	0.1	0.8	0.5
**Hypopharynx**	0.0	0.0	0.1	0.0	0.4	0.1	1.0	0.2	2.9	0.3	4.1	0.4	4.9	0.5
**Other Oral Cavity and Pharynx**	0.0	0.0	0.0	0.0	0.0	0.0	0.1	0.1	0.3	0.0	0.6	0.1	1.3	0.1
**Esophagus**	0.1	0.0	0.2	0.0	1.2	0.2	5.5	1.0	13.4	2.6	20.2	5.1	22.9	8.0
**Stomach**	0.5	0.6	1.9	2.2	6.8	5.1	17.1	10.0	41.6	21.5	86.9	44.9	125.8	74.9
**Small Intestine**	0.0	0.1	0.3	0.2	0.7	0.6	2.0	1.0	3.5	2.4	6.0	3.7	5.9	5.2
**Colon and Rectum**	1.5	1.4	6.6	5.8	23.8	18.8	72	50.3	133.1	82.2	202.3	134.8	241.5	206.3
**Anus, Anal Canal, and Anorectum**	0.1	0.0	0.1	0.1	0.5	0.4	0.7	0.9	1.2	1.1	1.7	2.3	2.0	2.8
**Liver**	0.5	0.2	3.2	0.7	13.3	2.8	37.7	7.7	68.1	22.7	88.2	42.9	88.1	50.6
**Gallbladder**	0.0	0.0	0.0	0.1	0.2	0.5	1.1	1.6	2.8	3.7	5.6	7.2	8.5	11.6
**Other Biliary**	0.0	0.0	0.1	0.2	0.9	0.6	2.7	1.7	8.1	4.7	15.7	8.8	20.3	17.0
**Pancreas**	0.1	0.2	0.9	0.7	3.5	2.6	11.6	7.8	30.4	22.2	54.0	46.8	82.0	76.0
**Retroperitoneum**	0.1	0.0	0.1	0.2	0.3	0.4	0.5	0.7	1.1	0.9	1.3	1.2	1.0	0.9
**Peritoneum, Omentum, and Mesentery**	0.0	0.0	0.0	0.1	0.1	0.4	0.1	0.9	0.2	2.3	0.1	3.3	0.2	1.9
**Other Digestive Organs**	0.0	0.0	0.1	0.0	0.2	0.2	0.8	0.6	1.5	1.0	2.7	1.9	6.8	5.2

**Nose, Nasal Cavity, and Middle Ear**	0.2	0.1	0.3	0.2	0.8	0.3	1.1	0.7	2.3	1.1	2.4	1.7	2.7	1.8
**Larynx**	0.0	0.0	0.2	0.0	0.8	0.1	3.5	0.4	8.0	1.1	12.1	1.3	14.2	1.3
**Lung Bronchus**	0.4	0.4	2.3	2.4	11.7	9.6	49.8	31.8	149.4	79.3	291.9	148.2	346.5	187.7
**Pleura**	0.0	0.0	0.0	0.0	0.0	0.0	0.0	0.0	0.0	0.0	0.1	0.1	0.2	0.1
**Trachea, Mediastinum, and Other Respiratory Organs**	0.5	0.0	0.2	0.1	0.2	0.1	0.2	0.1	0.3	0.1	0.2	0.2	0.4	0.3
**Soft Tissue Including Heart**	0.6	0.4	0.4	0.3	0.7	0.5	0.6	0.4	0.9	0.5	1.2	0.7	0.7	1.4
**Skin Excluding Basal and Squamous**	1.1	0.9	1.8	1.4	2.3	1.7	3.6	2.5	5.1	4.3	8.5	5.5	11.0	7.4
**Melanoma of the Skin**	0.3	0.4	0.7	1.0	1.0	1.4	2.1	1.8	3.9	2.2	5.3	3.3	6.4	5.0
**Other Non-Epithelial Skin**	0.3	0.3	0.5	0.6	0.7	0.7	1.0	0.9	1.7	1.3	4.2	1.8	6.9	4.7
**Urinary Bladder**	0.2	0.1	0.9	0.3	3.4	0.9	12.9	3.2	37.0	9.8	81.7	19.5	129.4	35.5
**Kidney and Renal Pelvis**	0.3	0.4	2.3	1.3	7.5	3.4	17.6	7.6	34.9	13.7	41.3	21.2	40.7	22.7
**Ureter**	0.0	0.0	0.0	0.0	0.1	0.0	0.3	0.1	1.5	0.9	3.9	2.4	5.1	3.8
**Other Urinary Organs**	0.0	0.0	0.0	0.0	0	0.0	0.2	0.1	0.6	0.4	1.2	0.6	2.2	1.0
**Eye and Orbit**	0.0	0.1	0.1	0.1	0.2	0.1	0.3	0.3	0.6	0.3	0.8	0.3	0.9	0.5
**Brain**	1.6	1.4	2.6	1.7	2.9	2.1	4.6	3.1	6.6	4.8	9.8	6.2	11.5	8.9
**Cranial Nerves Other Nervous System**	0.2	0.1	0.1	0.2	0.3	0.3	0.3	0.4	0.4	0.6	0.7	0.7	1.3	0.9
**Thyroid**	2.1	11.1	4.8	21.7	7.1	26.2	8.8	28.9	11.4	26.3	10.0	22.2	9.8	14.1
**Other Endocrine Including Thymus**	0.3	0.3	0.6	0.3	0.9	0.7	1.2	1.1	2.1	1.6	2.5	1.8	2.5	1.9
**Hodgkin Lymphoma**	2.3	2.4	1.3	1.4	1.0	0.8	1.1	0.7	1.4	0.7	2.3	1.3	2.6	1.4
**Non-Hodgkin Lymphoma**	3.0	2.2	5.1	3.8	10.0	6.8	19.8	14.6	42.4	26.7	67.5	48.4	90.9	63.2
**Myeloma**	0.1	0.0	0.5	0.2	1.5	1.3	5.8	4.1	13.4	8.7	24.0	17.1	26.7	18.3
**Lymphocytic Leukemia**	0.9	0.6	0.9	0.5	1.5	0.9	3.2	1.7	6.2	3.3	9.1	5.1	11.2	6.0

**Myeloid and Monocytic Leukemia**	1.9	1.4	2.6	1.9	3.8	2.7	5.3	4.2	11.0	6.8	22.2	11.5	30.7	18.7
**Other Leukemia**	0.1	0.0	0.2	0.2	0.2	0.1	0.4	0.3	1.1	0.7	2.5	1.7	5.7	4.0
**Mesothelioma**	0.0	0.0	0.0	0.1	0.1	0.1	0.4	0.2	1.5	0.4	3.3	1.0	4.5	0.7
**Kaposi Sarcoma**	0.3	0.0	1.5	0.0	1.5	0.0	0.5	0.0	0.5	0.0	0.5	0.1	1.0	0.3

Table S7. Male and female incidence rates per 100,000 by 10-year age groups among Latino individuals, SEER 2000-2019.

	20–29 years		30–39 years		40–49 years		50–59 years		60–69 years		70–79 years		80+ years	
**Cancer Site**	**Male rate**	**Female rate**	**Male rate**	**Female rate**	**Male rate**	**Female rate**	**Male rate**	**Female rate**	**Male rate**	**Female rate**	**Male rate**	**Female rate**	**Male rate**	**Female rate**
**Lip**	0.0	0.0	0.2	0.0	0.3	0.1	0.8	0.2	1.9	0.3	2.7	0.9	4.9	1.4
**Tongue**	0.1	0.1	0.3	0.3	1.2	0.9	4.4	1.5	7.6	3.1	8.8	3.7	7.4	5.3
**Salivary Gland**	0.2	0.3	0.4	0.4	0.7	0.8	1.1	1.1	2.3	1.6	4.2	2.5	6.8	3.0
**Floor of Mouth**	0.0	0.0	0.0	0.0	0.3	0.0	0.9	0.3	2.1	0.4	1.9	0.9	2.1	1.0
**Gum and Other Mouth**	0.1	0.1	0.2	0.2	0.6	0.3	1.6	0.8	3.4	1.7	4.5	3.7	4.9	5.7
**Nasopharynx**	0.1	0.1	0.2	0.2	0.5	0.2	1.0	0.4	1.3	0.6	1.7	0.7	1.2	0.8
**Tonsil**	0.0	0.0	0.1	0.0	1.4	0.2	4.1	0.9	6.0	1.1	5.6	1.4	3.1	0.9
**Oropharynx**	0.0	0.0	0.0	0.0	0.1	0.0	0.8	0.2	1.6	0.2	1.5	0.5	1.6	0.2
**Hypopharynx**	0.0	0.0	0.0	0.0	0.4	0.0	1.3	0.2	3.1	0.4	4.5	0.5	3.6	0.4
**Other Oral Cavity and Pharynx**	0.0	0.0	0.0	0.0	0.1	0.0	0.3	0.1	0.6	0.1	0.8	0.2	1.2	0.4
**Esophagus**	0.0	0.0	0.3	0.1	1.7	0.3	6.1	0.8	15.0	2.5	24.7	4.7	29.3	9.3
**Stomach**	0.4	0.5	2.5	2.5	6.5	6.0	16.5	11.3	37.6	20.9	72.4	40.3	97.7	61.0
**Small Intestine**	0.1	0.1	0.3	0.3	1.0	0.8	2.2	1.8	4.4	3.4	6.3	6.0	8.2	5.5
**Colon and Rectum**	1.2	1.4	5.2	5.0	17.3	15.7	58.8	43.8	122.7	77.8	187.3	128.0	228.9	192.5
**Anus, Anal Canal, and Anorectum**	0.0	0.0	0.3	0.1	1.0	0.7	1.7	2.3	2.2	3.9	2.7	4.8	3.1	6.8
**Liver**	0.2	0.2	0.7	0.4	6.9	1.6	34.8	7.5	65.6	21.3	74.7	39.8	71.8	42.8
**Gallbladder**	0.0	0.0	0.1	0.2	0.4	1.5	1.1	3.9	3.4	9.6	6.6	16.1	9.7	19.8
**Other Biliary**	0.0	0.0	0.2	0.2	0.9	0.6	2.9	2.4	7.5	6.1	14.9	10.3	19.0	17.3
**Pancreas**	0.1	0.2	0.7	0.6	3.4	2.8	12.1	9.3	33.6	26.4	59.1	52.2	72.4	82.1
**Retroperitoneum**	0.1	0.0	0.1	0.1	0.3	0.3	0.6	0.8	1.1	0.8	1.6	1.1	1.4	0.9
**Peritoneum, Omentum, and Mesentery**	0.0	0.0	0.0	0.1	0.1	0.3	0.1	1.1	0.2	2.9	0.7	4.3	0.4	2.9
**Other Digestive Organs**	0.0	0.0	0.1	0.1	0.3	0.3	0.8	0.6	1.7	1.7	3.6	3.0	6.1	6.7

**Nose, Nasal Cavity, and Middle Ear**	0.1	0.1	0.2	0.2	0.6	0.3	1.0	0.7	1.8	1.2	2.7	1.5	3.0	2.5
**Larynx**	0.0	0.0	0.2	0.1	1.4	0.3	5.8	0.8	14.2	1.7	24.3	2.9	20.1	2.2
**Lung Bronchus**	0.3	0.4	1.2	1.2	5.7	5.8	26.6	21.3	97.6	63.2	216.9	129.9	271.6	151.6
**Pleura**	0.0	0.0	0.0	0.0	0.0	0.0	0.0	0.0	0.1	0.0	0.3	0.1	0.6	0.1
**Trachea, Mediastinum, and Other Respiratory Organs**	0.5	0.0	0.3	0.0	0.2	0.1	0.1	0.0	0.3	0.2	0.4	0.3	0.1	0.2
**Soft Tissue Including Heart**	0.9	0.5	0.6	0.5	0.7	0.6	0.9	0.7	1.0	0.9	1.7	1.0	1.6	1.5
**Skin Excluding Basal and Squamous**	1.2	1.2	1.9	1.7	2.4	2.3	4.2	3.3	6.4	4.7	10.7	6.8	11.2	8.1
**Melanoma of the Skin**	0.5	1.3	1.4	3.3	3.0	4.9	5.8	7.1	11.4	9.7	16.6	12.6	22.1	14.7
**Other Non-Epithelial Skin**	0.3	0.4	0.6	0.6	0.8	0.8	1.1	1.0	1.9	1.9	4.4	3.4	7.9	5.2
**Urinary Bladder**	0.2	0.2	0.9	0.5	3.2	1.2	13.0	3.7	42.2	11.8	89.8	23.8	144.8	41.1
**Kidney and Renal Pelvis**	0.5	0.5	3.1	2.7	11.8	7.5	28.0	16.6	56.6	30.4	70.3	39.9	63.8	39.3
**Ureter**	0.0	0.0	0.0	0.0	0.0	0.0	0.2	0.1	0.9	0.4	2.1	1.2	3.3	2.3
**Other Urinary Organs**	0.0	0.0	0.0	0.0	0.1	0.0	0.2	0.1	0.7	0.3	1.4	0.5	2.9	1.1
**Eye and Orbit**	0.1	0.1	0.2	0.1	0.3	0.2	0.6	0.6	1.3	1.0	2.2	1.2	2.8	1.6
**Brain**	1.7	1.5	2.6	2.3	4.2	3.0	6.8	5.2	11.6	8.3	17.4	12.1	16.3	13.7
**Cranial Nerves Other Nervous System**	0.2	0.1	0.3	0.2	0.4	0.3	0.5	0.4	0.3	0.6	0.8	0.9	0.8	0.7
**Thyroid**	1.6	10.5	3.2	18.4	5.1	24.1	7.7	26.6	9.1	25.7	9.7	23.1	8.1	13.4
**Other Endocrine Including Thymus**	0.2	0.2	0.3	0.2	0.4	0.4	0.7	0.8	1.3	1.2	2.1	1.2	1.8	1.1
**Hodgkin Lymphoma**	2.4	2.6	2.2	1.9	2.3	1.3	3.1	1.8	4.1	2.8	7.2	3.8	5.5	3.5
**Non-Hodgkin Lymphoma**	3.2	1.9	6.8	4.0	12.9	8.3	24.8	19.0	47.4	38.0	78.3	64.7	92.2	76.9
**Myeloma**	0.1	0.1	0.7	0.4	2.7	2.0	8.4	6.3	20.0	14.8	34.4	24.9	41.3	26.5
**Lymphocytic Leukemia**	1.8	1.0	1.6	1.0	2.3	1.5	4.7	3.4	10.2	7.2	17.8	10.6	24.1	13.7

**Myeloid and Monocytic Leukemia**	1.9	1.4	2.6	1.9	3.6	3.0	5.7	4.6	10.9	8.0	20.8	13.6	32.1	18.7
**Other Leukemia**	0.2	0.1	0.2	0.1	0.2	0.2	0.6	0.4	1.0	0.7	3.2	1.8	5.9	4.8
**Mesothelioma**	0.0	0.0	0.1	0.1	0.4	0.3	1.1	0.4	3.6	0.9	9.4	2.6	14.6	3.2
**Kaposi Sarcoma**	1.7	0.0	6.0	0.1	4.3	0.1	2.5	0.1	1.7	0.3	2.8	1.0	4.5	2.3

Table S8. Male and female incidence rates per 100,000 by 10-year age groups among American Indian/Alaska Native individuals, SEER 2000-2019.

	**20–29 years**		**30–39 years**		**40–49 years**		**50–59 years**		**60–69 years**		**70–79 years**		**80+ years**	
**Cancer Site**	**Male rate**	**Female rate**	**Male rate**	**Female rate**	**Male rate**	**Female rate**	**Male rate**	**Female rate**	**Male rate**	**Female rate**	**Male rate**	**Female rate**	**Male rate**	**Female rate**
**Lip**	0.0	0.0	0.0	0.0	0.5	0.3	0.8	0.0	3.3	0.3	3.9	0.6	6.9	2.2
**Tongue**	0.0	0.1	0.4	0.0	2.7	0.9	4.9	3.1	10.0	3.3	8.9	2.8	2.1	2.2
**Salivary Gland**	0.3	1.0	1.2	1.2	1.0	1.3	1.0	2.2	1.8	1.5	3.7	2.3	7.6	1.1
**Floor of Mouth**	0.0	0.0	0.0	0.1	0.6	0.3	2.0	1.6	5.4	0.3	2.8	1.1	3.8	0.0
**Gum and Other Mouth**	0.1	0.0	0.6	0.1	0.6	0.3	3.3	0.5	4.2	3.5	3.7	3.8	5.2	5.6
**Nasopharynx**	0.4	0.1	1.9	0.4	3.3	0.6	6.0	2.6	10.9	5.6	10.0	4.7	9.4	3.4
**Tonsil**	0.0	0.0	0.3	0.0	1.4	0.3	5.4	1.1	7.7	1.2	3.7	0.6	2.1	1.1
**Oropharynx**	0.0	0.0	0.0	0.0	0.6	0.1	1.4	0.6	1.9	0.3	1.3	0.5	1.7	1.1
**Hypopharynx**	0.0	0.0	0.0	0.0	0.2	0.1	2.7	1.3	6.3	0.6	8.3	1.0	7.6	0.0
**Other Oral Cavity and Pharynx**	0.0	0.0	0.0	0.0	0.2	0.1	1.1	0.2	1.0	0.3	0.0	0.0	3.8	1.1
**Esophagus**	0.1	0.0	0.2	0.1	2.7	0.9	11.3	3.5	23.1	6.1	34.0	10.6	50.2	14.5
**Stomach**	0.4	0.9	4.7	5.8	10.7	5.3	24.0	13.5	51.3	25.5	92.2	41.1	114.9	65.0
**Small Intestine**	0.1	0.1	0.3	0.4	0.5	1.5	2.0	1.9	7.7	3.2	7.4	4.0	5.5	4.5
**Colon and Rectum**	2.3	2.5	11.3	10.3	31.3	31.2	89.1	72.1	172.8	126.4	242.2	225.5	292.6	254.2
**Anus, Anal Canal, and Anorectum**	0.0	0.0	0.2	0.4	1.4	1.6	2.5	2.5	4.7	3.9	0.9	3.1	3.8	6.7
**Liver**	0.6	0.4	1.5	1.1	7.1	4.0	42.0	13.0	67.5	30.4	75.9	50.1	97.3	61.6
**Gallbladder**	0.0	0.1	0.0	0.2	0.6	1.5	1.7	6.1	9.8	12.9	17.9	23.7	30.2	39.2
**Other Biliary**	0.0	0.0	0.0	0.1	1.4	0.4	2.4	2.4	10.9	6.8	18.5	18.7	21.2	23.5
**Pancreas**	0.1	0.0	0.4	0.8	5.4	3.4	17.3	15.5	44.0	26.2	67.6	51.1	85.1	87.4
**Retroperitoneum**	0.1	0.1	0.2	0.1	0.2	0.0	0.4	0.7	0.0	1.2	0.7	0.6	0.0	0.0
**Peritoneum, Omentum, and Mesentery**	0.0	0.0	0.0	0.1	0.0	0.1	0.2	0.4	0.4	2.7	0.0	1.1	0.0	2.2
**Other Digestive Organs**	0.0	0.0	0.2	0.0	0.6	0.3	2.1	1.6	4.1	4.0	5.2	6.9	11.8	12.3

**Nose, Nasal Cavity, and Middle Ear**	0.2	0.0	0.5	0.3	0.6	0.9	0.8	0.8	2.1	0.9	3.1	2.8	10.7	7.8
**Larynx**	0.0	0.0	0.0	0.0	2.1	0.4	8.7	1.1	14.1	3.8	22.7	2.0	13.2	2.2
**Lung Bronchus**	0.5	0.2	2.0	0.7	11.6	10.0	60.5	44.5	175.9	121.7	370.3	214.5	361.5	207.4
**Pleura**	0.0	0.0	0.0	0.0	0.0	0.0	0.0	0.0	0.6	0.0	0.0	0.0	0.0	0.0
**Trachea, Mediastinum, and Other Respiratory Organs**	0.2	0.0	0.0	0.0	0.2	0.0	0.2	0.0	0.4	0.0	0.7	0.0	1.7	0.0
**Soft Tissue Including Heart**	0.7	1.0	0.8	0.7	0.9	0.7	0.4	1.1	0.7	1.1	2.0	2.0	1.7	2.2
**Skin Excluding Basal and Squamous**	2.3	1.4	2.4	1.4	3.2	2.2	3.5	4.5	5.8	2.6	9.6	7.4	13.9	9.0
**Melanoma of the Skin**	0.1	2.7	2.9	4.0	2.9	5.1	6.6	5.1	10.5	9.6	17.2	10.0	21.4	21.3
**Other Non-Epithelial Skin**	0.1	1.0	0.7	1.1	1.0	0.9	1.0	1.0	2.8	2.3	4.6	6.0	5.9	4.5
**Urinary Bladder**	0.3	0.6	1.9	0.1	3.6	1.6	15.7	3.9	40.4	9.2	64.3	14.3	115.4	20.2
**Kidney and Renal Pelvis**	0.8	1.0	8.0	4.4	22.1	12.4	46.5	27.2	89.5	48.0	132.1	54.2	118.3	51.6
**Ureter**	0.0	0.0	0.0	0.0	0.0	0.0	0.6	0.0	0.7	0.3	1.7	0.5	0.0	0.0
**Other Urinary Organs**	0.0	0.0	0.0	0.0	0.0	0.0	0.2	0.0	0.7	0.3	2.4	0.0	0.0	1.1
**Eye and Orbit**	0.1	0.1	0.2	0.3	0.0	0.2	0.2	0.4	0.4	0.9	1.3	1.1	3.8	3.4
**Brain**	2.5	1.7	3.6	2.9	3.7	2.5	4.1	3.5	9.2	4.4	14.0	5.8	14.9	5.6
**Cranial Nerves Other Nervous System**	0.3	0.4	0.3	0.3	0.0	0.7	0.8	0.2	1.0	1.0	0.0	0.0	0.0	0.0
**Thyroid**	1.8	12.4	3.4	22.7	4.6	21.5	6.9	24.3	12.6	20.1	8.3	20.4	5.9	19.1
**Other Endocrine Including Thymus**	0.6	0.1	0.3	0.4	0.2	0.1	0.8	0.4	1.0	1.1	1.5	0.0	0.0	0.0
**Hodgkin Lymphoma**	1.4	1.2	1.6	1.8	1.3	0.2	1.4	0.9	2.0	1.7	0.9	4.0	3.8	3.4
**Non-Hodgkin Lymphoma**	1.2	1.4	5.9	4.4	9.6	7.7	21.1	21.1	39.4	31.4	57.8	42.3	66.0	66.1
**Myeloma**	0.3	0.1	1.2	0.6	3.7	2.6	7.7	8.6	18.7	18.1	35.8	33.6	38.5	25.8
**Lymphocytic Leukemia**	1.8	1.3	1.3	1.6	0.8	0.9	6.2	4.4	8.5	4.1	13.7	6.9	16.6	7.8

**Myeloid and Monocytic Leukemia**	2.1	1.1	3.3	2.8	1.9	3.1	8.7	5.0	11.0	6.3	14.4	10.6	22.6	3.4
**Other Leukemia**	0.4	0.1	0.1	0.1	0.5	0.7	1.8	0.9	2.6	1.0	2.2	2.8	5.9	2.2
**Mesothelioma**	0.1	0.0	0.3	0.0	0.6	0.2	1.4	0.9	3.6	0.3	4.8	2.9	14.9	4.5
**Kaposi Sarcoma**	1.0	0.0	2.6	0.0	2.4	0.0	1.3	0.0	1.4	0.0	2.4	1.1	3.5	3.4
